# Endoscopic full-thickness resection: a minimally invasive approach for jejunal submucosal tumors

**DOI:** 10.1055/a-2590-1802

**Published:** 2025-05-14

**Authors:** Yi Chen, Wenhui Xia, Qianqian Wang, Yuxuan Chen, Jianyu Lv, Shuo Zhang

**Affiliations:** 1 587400 Department of Gastroenterology, The Second Affiliated Hospital of Zhejiang Chinese Medical University, Hangzhou City, Zhejiang Province, China

**Fig. 1:**
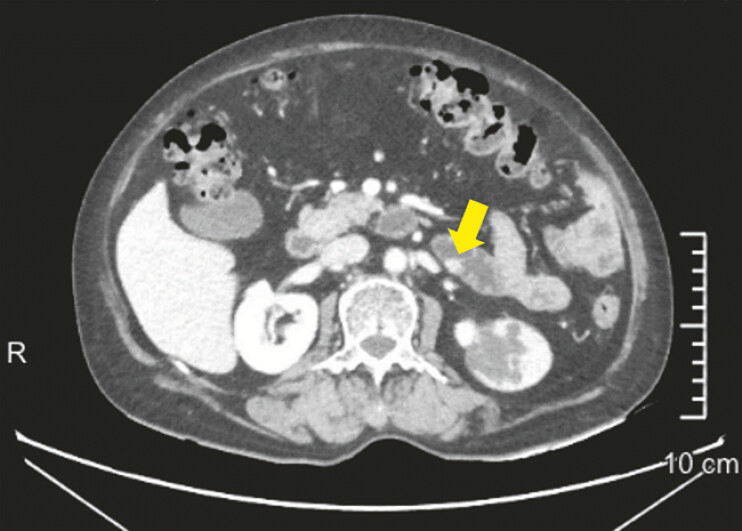
Enhanced CT of the upper abdomen showed a blood-rich nodule on the posterior wall of the proximal jejunum.

**Fig. 2:**
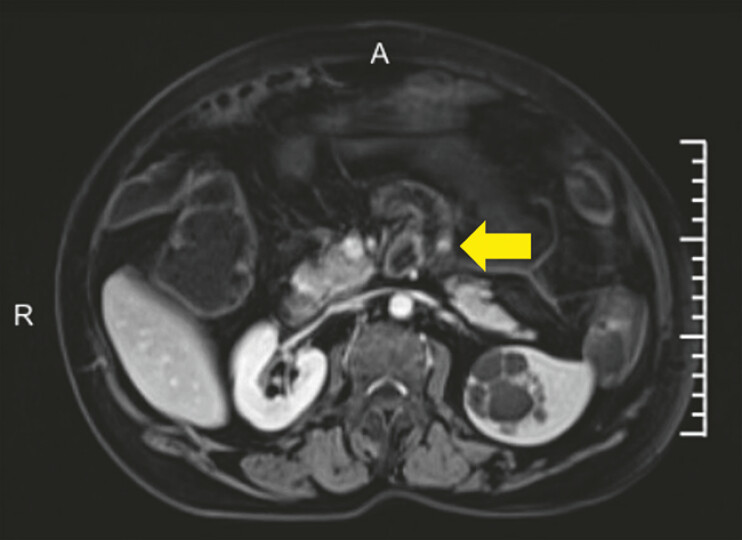
MRI showing abnormal signal nodules in the proximal jejunum.

**Fig. 3:**
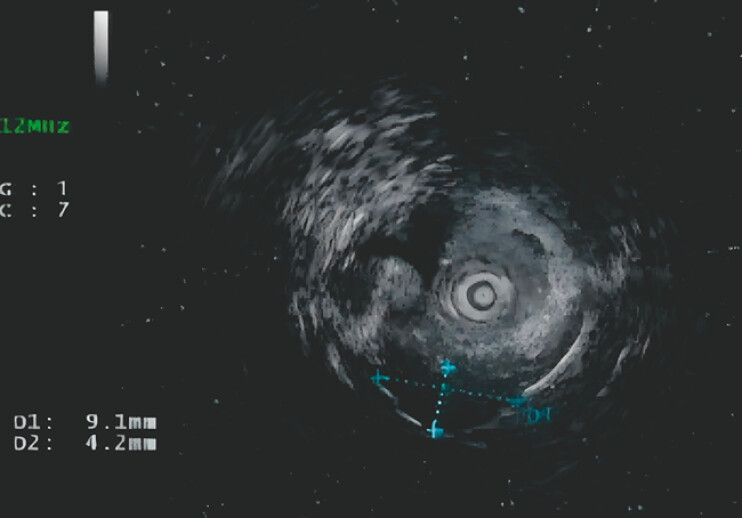
Ultrasound colonoscopy showed a submucosal bulge with a maximum cross-section of approximately 9.1 mm × 4.2 mm.

**Fig. 4:**
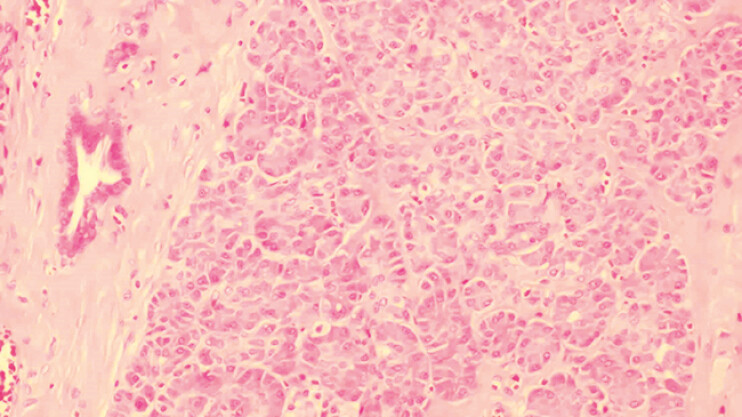
Pathology showed 1.3 × 1 × 0.8 cm ectopic pancreatic tissue.

A 63-year-old woman was found with a blood-rich nodule on the posterior wall of the proximal jejunum during an enhanced abdominal CT ( Fig. 1 ). The patient was asymptomatic and had nothing unusual in physical examination or laboratory tests. Enhanced MR also suggests an abnormal signal nodule ( Fig. 2 ). Colonoscopy reveals a submucosal bulge in the upper jejunum. Further endoscopic ultrasonography showed a homogeneous hypoechoic interior of the lesion, originating in the lamina propria ( Fig. 3 ). Because of the tumor’s position within the deep muscularis propria and near the serosal layer, full-thickness resection was performed ( Video 1 ). The lesion measures approximately 13 mm × 10 mm × 8 mm in vitro. The pathological diagnosis was ectopic pancreatic tissue ( Fig. 4 ). The procedure went successfully. Postoperatively, the patient recovered well with no adverse events.

Unlocking the potential of EFTR: minimally invasive solutions for jejunal submucosal tumors.Video 1

Management of small bowel masses is considered problematic. Although endoscopic treatment offers minimally invasive options, the difficulty of maneuver remains a great challenge. We are the first to successfully implement the EFTR technique for the complete removal of jejunal tumors. Despite the artificial perforation inevitably associated with total resections, the innovation and application of endoscopic closure technology promotes mucosal healing, enhances the safety of the procedure and broadens the indications for EFTR.

Endoscopy_UCTN_Code_TTT_1AP_2AD

